# Rapid breeding and varietal replacement are critical to adaptation of cropping systems in the developing world to climate change

**DOI:** 10.1016/j.gfs.2017.01.008

**Published:** 2017-03

**Authors:** Gary N. Atlin, Jill E. Cairns, Biswanath Das

**Affiliations:** a Bill & Melinda Gates Foundation, United States; b CIMMYT, Zimbabwe; c Syngenta, Zambia

**Keywords:** Climate change adaptation, Rapid crop breeding, Varietal replacement, Germplasm exchange, Genetic gains, Seed systems

## Abstract

Plant breeding is a key mechanism for adaptation of cropping systems to climate change. Much discussion of breeding for climate change focuses on genes with large effects on heat and drought tolerance, but phenology and stress tolerance are highly polygenic. Adaptation will therefore mainly result from continually adjusting allele frequencies at many loci through rapid-cycle breeding that delivers a steady stream of incrementally improved cultivars. This will require access to elite germplasm from other regions, shortened breeding cycles, and multi-location testing systems that adequately sample the target population of environments. The objective of breeding and seed systems serving smallholder farmers should be to ensure that they use varieties developed in the last 10 years. Rapid varietal turnover must be supported by active dissemination of new varieties, and active withdrawal of obsolete ones. Commercial seed systems in temperate regions achieve this through competitive seed markets, but in the developing world, most crops are not served by competitive commercial seed systems, and many varieties date from the end of the Green Revolution (the late 1970s, when the second generation of modern rice and wheat varieties had been widely adopted). These obsolete varieties were developed in a climate different than today's, placing farmers at risk. To reduce this risk, a strengthened breeding system is needed, with freer international exchange of elite varieties, short breeding cycles, high selection intensity, wide-scale phenotyping, and accurate selection supported by genomic technology. Governments need to incentivize varietal release and dissemination systems to continuously replace obsolete varieties.

## Highlights

• Continuous turnover of crop varieties is critical to climate change adaptation.
• Farmers in commercial temperate cropping systems, with rapid varietal replacement, are best protected.
• Breeding programs need unfettered access to elite varieties from regions now facing their future climate.
• Rapid and effective breeding cycles are needed to deliver climate change adaptation in real time.
• Farmers in the developing world are at risk from slow breeding and varietal replacement cycles.

## 1 Introduction: the challenge posed by climate change for crop production, and the problem of obsolete varieties

In food-insecure regions in Africa, climate change is expected to reduce yields through increased average temperatures (Cairns et al., 2013a, Knox et al., 2012, Challinor et al., 2016), and increased frequency of extreme weather events (Lesk et al. 2016). High temperatures during and after flowering reduce grain set in wheat (Stratonovitch and Semenov, 2015), and biomass accumulation and seed-set in rice (Peng et al., 2004). They accelerate development in maize and senescence in wheat, reducing yield potential (Challinor et al., 2016, Lobell et al., 2012, Asseng et al., 2015). Maize yield losses average approximately 1% for each growing degree day (GDD) above 30 C in sub-Saharan Africa (Lobell et al., 2011), and also result from extreme heat events (Lobell et al., 2013). Climate change also affects pest and disease prevalence (Dawson et al., 2015).

Concerns about such effects have prompted widespread efforts to identify major genes affecting drought, flooding, and heat tolerance. Some alleles with large effects on these traits have been identified ((e.g., in rice, Xu et al., 2006: Bernier et al., 2007; Ye et al., 2015), but they are relatively rare, and will likely provide only a small portion of the genetic variability needed for adaptation. Adaptation will be achieved by: matching phenology to growing season length through changes in cultivar day-length and temperature response (Kumudini et al., 2014), exemplified by the shift from photoperiod-sensitive landraces to photoperiod-insensitive semi-dwarf wheat and rice varieties during the Green Revolution, and changes in root architecture allowing better access to soil water (Lynch and Wojciechowski, 2015); transpiration response to high vapor pressure deficit (Messina et al., 2015); and cellular processes affecting heat and desiccation tolerance (Mickelbart et al., 2015). The genetic architecture of these traits tends to be highly polygenic. Even control of flowering time has been shown to be influenced by many genes with small effects (e.g. Buckler et al., 2009).

It is also unclear that widespread varietal adoption can be driven by major genes for stress tolerance. For example, incorporation of the highly effective Sub1 allele for submergence tolerance in rice has led to only limited adoption of Sub1 varieties except in areas where flash flooding is frequent and severe (IRRI, unpublished data). Farmers adopt new varieties based on many considerations, notably yield potential, end-use quality, and agronomic fit to their cropping system. Large-effect alleles for stress tolerance must be packaged in varieties that are profitable to produce and demanded by end-users (who, in developing countries, usually include the farmers themselves). Cropping system adaptation to climate change will therefore mainly result from breeding programs that deliver continuous optimization of quantitatively inherited trait complexes, requiring constant and rapid gene frequency change in elite populations, and seed systems that continuously deploy the improved cultivars extracted from these populations. The farmers who are best protected from climate change are those who have access to a steady stream of new cultivars bred in the current climate. Farmers in many temperate regions have this access, due to competitive seed sectors that encourage varietal turnover. In contrast, most farmers in climate-vulnerable areas of the developing world use improved cultivars selected thirty or more years ago, or landraces selected generations ago, in a different climate. To increase yields in these regions in the face of climate change, increased investment in accelerated breeding and varietal dissemination is urgently needed, as is access to elite germplasm from regions already experiencing the “future climate”.

This paper will argue that the key elements of cropping system adaptation to climate change are:

(i) Access to elite germplasm from other regions that currently experience conditions likely to occur in the target region as a result of climate change;
(ii) Rapid breeding cycles that provide farmers with a steady stream of new cultivars developed in and for the current climate;
(iii) Evaluation of potential new cultivars under the full range of climate conditions they are likely to encounter over their commercial life;
(iv) Seed systems that deliver new varieties to farmers quickly, and then just as quickly replace them, keeping pace with the changing climate.

These elements characterize highly commercialized systems in temperate regions, but are not in place in the developing world. Consequently, smallholder farmers in developing countries are at much greater risk from climate change than farmers in richer regions. Plant breeding and seed systems in the developing world must be rapidly upgraded to protect vulnerable farmers. Of course, improved plant breeding and varietal replacement systems are only part of the toolkit needed to deliver climate change adaptation. Especially in Africa, improvements in soil fertility management are urgently required. On a continent where the average inorganic N, P_2_O_5_, and K_2_O fertilizer application rates on cropland were only 13.8, 5.9, and 2.2 kg ha^−1^, respectively, in 2014 (FAO, 2015), or about one-sixth of the global average, yield losses for the foreseeable future due to climate change could be more than counterbalanced by bringing fertilizer use closer to the global mean. Smallholder farmers in the developing world need a host of supports to intensify production, including secure land tenure, improved market access, credit, and transportation infrastructure. However, this review will focus on the improved cultivar development and dissemination systems that are needed to quickly develop and deliver the shorter-duration, stress-tolerant, market-demanded, higher-yielding varieties demanded by small-holder farmers who are intensifying production in the face of a rapidly changing climate. In most countries severely affected by climate change, the systems for delivering these adaptation tools are inadequate.

## 2 Elements of climate-adaptive breeding and seed systems

### 2.1 Access to elite germplasm and performance data from other regions: the critical role of international public breeding programs

Most of the tools needed for adaptation are already in our hands. For most crop species, tolerance to the range of variability in predicted temperatures and precipitation over the next 100 years lies within the current genetic diversity (Burke et al., 2009, Braun et al., 2010). Little of this variability has been deployed as elite cultivars in the regions that are both most food insecure and most vulnerable to climate change. Elite cultivars from regions already experiencing the expected climate for the breeding target region, which are easier to use as direct parents in breeding than landraces, will often have to be acquired from beyond national borders (Galluzi et al., 2015). Unfortunately, the open culture of germplasm exchange existing until about 25 years ago has become restrictive (Galluzi et al., 2015; Heisey and Day Rubenstein, 2015). Until the 1990s, breeders exchanged varieties relatively freely. However, as plant breeding became highly commercialized, companies increasingly sought IP protection for products, culminating in the US practice of protecting cultivars with utility patents that prevent them from being used as parents by other breeders. At about the same time, many countries recognized their indigenous crop genetic resources as a unique patrimony, and restricted their international exchange. Any breeder who has recently attempted to obtain an elite variety from the national system of a different country knows that this has become very difficult. There is little “freedom to operate” for the public sector plant breeding programs that serve most smallholder farmers in developing countries.

What of the plant genetic resources collections of national agricultural systems and the international crop research institutes of the Consultative Group on International Agricultural Research (CGIAR)? CGIAR gene banks, as well as those of the USDA, still freely provide breeding programs with access to the great diversity in their collections (Heisey and Day Rubenstein, 2015, Galluzi et al., 2015). However, these collections consist mainly of unimproved landraces, which are important sources of alleles for stress tolerance and disease resistance but are usually narrowly adapted to their environment of origin, and are unsuitable for modern commercial agriculture because they lack the fertilizer responsiveness and yield potential farmers need now. Most breeding programs cannot afford to use unimproved gene bank accessions directly as parents in breeding for greater heat and drought tolerance, due to the yield penalty usually associated with them. Although inexpensive molecular marker systems are reducing the cost of localizing and exploiting genes for stress tolerance in unimproved materials, breeders still have great need of elite, commercially-acceptable materials from regions already experiencing the expected climate. Elite varieties from warmer, drier, or wetter regions are the critical building materials needed to construct varieties adapted to the future climate in their own target regions. Some public germplasm collections also acquire and maintain older elite improved varieties, but the vast majority of their improved holdings were developed over 30 years ago, in a different climate and under different agricultural conditions.

How, then, can breeders of the staple crops in the developing world acquire the elite germplasm needed to rapidly adapt to a changing climate? Multinational seed companies, which focus on highly commercialized crops like maize and soy, often have operations or alliances in different countries, and can move their elite proprietary germplasm among these countries. On the other hand, small national programs and regional seed companies usually lack the international connections needed to cope with a rapidly changing climate, and sharing of germplasm between national programs in different countries is limited and difficult. Of more direct use are the new elite materials continually being generated by CGIAR breeding programs, which focus strongly on developing commercial varieties with improved heat, flooding, and drought tolerance that are freely available to, and widely used by, national programs and seed companies (eg Cairns et al., 2013b). The preservation and expansion of the CGIAR's global breeding networks is critical to the developing world's capacity to adapt to climate change. These networks, which often test varieties in a broad range of environments and regions, generating materials with a broad range of adaptation, are often the only accessible sources of elite germplasm from other countries for small breeding programs. They are critical to climate change adaptation in the developing world.

### 2.2 The importance of rapid breeding cycles

Although climate change is occurring rapidly, it is still relatively gradual in historical terms. The best predictor of the climate in the very near future, (i.e. the next ten years) is the current climate. Farmers who are at least risk with respect to climate change are those who use varieties bred very recently. The most important climate change adaptation tools for crop production are thus breeding and cultivar delivery systems that rapidly and continuously develop new varieties and replace old ones. Farmers working in cropping systems served by highly competitive commercial seed industries, e.g. maize and soybean producers in the US Corn Belt, are served by such systems. Most farmers in Sub-Saharan Africa and South Asia are not; Challinor et al. (2016) noted that the time to develop and deliver maize varieties in sub-Saharan Africa is currently around 30 years, a period in which substantial climate change has occurred. Farmers in much of the developing world are using varieties selected in a rather different climate.

**Fig. 1:**
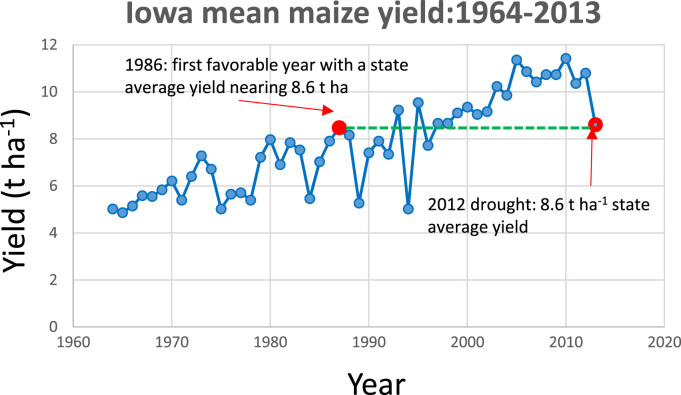
Iowa maize yields from 1964 to 2013, showing that the reduced yield in the severe drought year of 2012 was equivalent to a high yield in the 1980s.

**Fig. 2:**
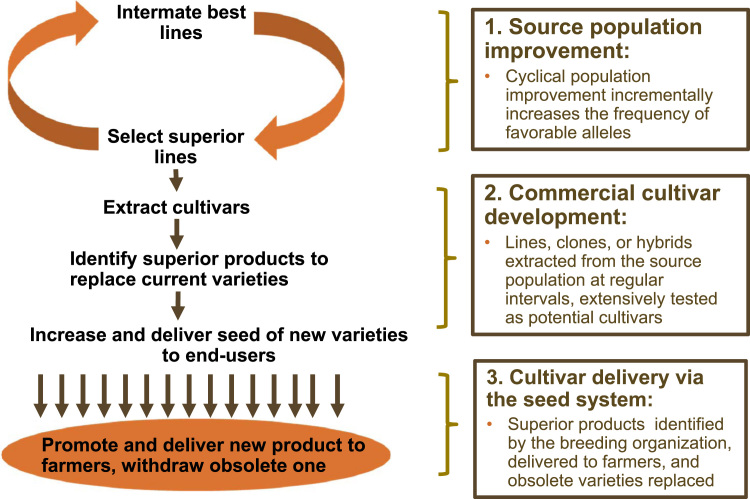
: Post-Green Revolution crop improvement is a continuous, cyclical process that gradually improves populations from which varieties are selected and delivered to farmers via the seed system.

The power of rapid-cycle breeding to drive climate change adaptation is illustrated in Fig. 1, which presents maize yields from the US state of Iowa (the heart of the US Corn Belt) for 50 years through 2013. From 1980 through 2013, growing season average temperature has increased by 0.15 C per decade, with decreased precipitation in the late growing season (Dai et al., 2015). Both trends are unfavorable for maize yield, but yields increased at an annual rate of 1.51% (142 kg ha^−1^) during this period. 2012 was the worst drought year in Iowa since 1988 (Boyer et al., 2013). Yet the mean maize yield for the state in that year was 8.80 t ha^−1^, a yield not reached in a favorable year until 1986, and not routinely achieved until the late 1990s. This is the result of rapid and effective maize breeding and seed delivery, driving yield improvement in the face of climate change. Commercial maize breeding in the US is a case study in how to design a plant breeding and seed system that delivers genetic gains and climate change adaptation. The key features of such a system can be identified by analyzing the three main components of the plant breeding and dissemination process (Fig. 2):

1) *Population improvement,* wherein segregating populations are formed, recombinants with high phenotypic and, presumably, genotypic value selected, and the selected recombinants intermated to form a new cycle of the breeding population, incrementally improved over the previous cycle as a result of changed allele frequencies. It is this component of the breeding system that drives climate change adaptation.
2) *Commercial cultivar selection,* wherein selected lines, families, or clones (usually the same as those selected for recombination) are subjected to further evaluation and selection to determine their potential for release to farmers as cultivars.
3) *Release and dissemination of new cultivars, and replacement of obsolete ones,* wherein candidate cultivars superior to currently-used varieties are scaled up and disseminated to farmers, with obsolete cultivars withdrawn from production.

Rapid breeding cycles (step 1 above) drive climate change adaptation by changing allele frequencies in breeding populations; the commercial cultivar selection and release and dissemination steps are critical in determining how effectively this adaptation is delivered to farmers, but they do not drive allele frequency change in source populations until new lines are used as parents of the next breeding cycle. Rapid breeding cycles are particularly important in developing country cropping systems that are often rapidly intensifying and subject to progressive soil fertility decline (Vanlaue et al. 2015). A rapid-cycle cultivar development, testing, and deployment system can provide cropping systems with adaptation to climate change by constantly generating incrementally changed allelic combinations and subjecting these combinations to intense selection pressure in the target population of environments (TPE). But the vast majority of breeding programs serving farmers in the developing world are cycling their breeding populations too slowly. Whereas the best private-sector programs have cycle times of three to four years from the generation of a new breeding population until the best lines selected from it are themselves used as parents of new populations, public sector breeding programs in developing countries usually have very long breeding cycles. They often take over 10 years to develop and evaluate a new variety to the point where they feel confident in “recycling” it as a parent, and even longer to deploy it. If the accuracy and intensity of selection is constant, then halving cycle time will double the rate of genetic gain. Cutting cycle time has the added benefit of increasing the frequency with which haplotypes are recombined and exposed for selection in the constantly changing environment, increasing the probability of creating and selecting allelic combinations that are closer to optimal for current conditions. Rapid breeding cycles are also critical for adaptation to evolving pest and pathogen populations.

Breeding program optimization based on sound quantitative genetic principles can significantly shorten breeding cycles, by reducing excessive testing before selection of parents. Both biometric theory and experimental variance component analyses in many species show clearly that most of the accuracy in the estimation of genotypic or breeding value results from testing in a modest but representative set of environments over one or two years (e.g. Atlin et al., 2000). Experience with public sector breeders in the developing world indicates that excessive testing of promising new breeding lines before they are recycled as parents is a very common error. Adoption of breeding tools such as rapid-generation advance in off-season nurseries or controlled environments, doubled-haploid technology, and genomic selection ((O’Connor et al. 2013; Prigge et al., 2011; Heffner et al., 2010) can also cut years from the breeding cycle. The importance of these tools in reducing breeding cycle time is illustrated in Table 1, which compares cycle length for typical breeding plans for self-pollinated crops like rice, wheat, and beans. The most common breeding scheme used in the developing world is pedigree breeding without off-season generation advance, and with at least three years of yield testing before new lines are used as parents for the next cycle. This is typically a ten- to twelve-year process. The breeding cycle length can be reduced by 30% simply by adding an off-season generation advance, which in many tropical regions requires only modest investment in irrigation. If the number of years of testing prior to use as a parent is reduced as well, cycle times can be halved. Using the full suite of breeding acceleration techniques can reduce the breeding cycle to three or four years from ten in most species. This degree of acceleration, already achieved in maize, soy, barley, rapeseed, and wheat in North America and Western Europe, is ample for keeping pace with a changing climate.

**Table 1:** Breeding cycle times for several breeding schemes.

Line development scheme	Testing scheme		
	Conventional 3-year replicated phenotyping before use of new lines as parents	Accelerated 2-year replicated phenotyping before use of new lines as parents	Single-year phenotyping plus genomic selection
	Total breeding cycle time (years) assuming lines are inbred to F_6_ generation (or complete homozygosity for doubled haploid systems)		
Pedigree, no off-season nursery	10	9	8
Pedigree with off-season nursery	7	6	5
Single-seed descent in controlled environment	6	5	4
Doubled haploid	5	4	3

### 2.3 Evaluation of potential new varieties in a wide range of environments: the need for multi-location testing networks

Year-to-year and location-to-location variation in the amount and timing of rainfall, as well as in the occurrence and severity of extreme temperature events, is substantial in many crop-producing regions, and may be increasing as a result of climate change (Stratonovitch and Semenov, 2015). The annual weather variability at any particular location can be greater than the variation in long-term average temperature or rainfall at individual locations within a breeding program's target region. Therefore, although crop cultivars are bred in and for specific regions, they need to be adapted to weather variability within those regions, both within and across years. Cultivars developed by multinational seed companies and international crop research centers are evaluated across many locations and several years during development to ensure that they are exposed to a representative sample of the TPE they will encounter in farmers’ fields (Cooper and Delacy, 1994, Cooper et al., 2014). Recently, commercial drought-tolerant hybrids were evaluated in over 10,000 trials in the Corn Belt during advanced selection (Gaffney et al., 2015). As a result of this extensive evaluation, they exhibit very broad adaptation to the range of environmental and management conditions that occur within the TPE. This can translate into adaptation even to regions where the cultivar was not originally tested, because similar production environments can occur across regions and even continents. For example, Kebede et al. (2013) have shown that maize variety trials conducted in subtropical areas of Mexico and southern Africa yield highly correlated results. The similarity of production environments across vast regions has led to the selection of varieties exhibiting very broad adaptation to a range of climatic conditions. For example, the maize inbred line CML312 has contributed to hybrids grown throughout Latin America and sub-Saharan Africa. Early Green Revolution wheat and rice varieties were grown on millions of hectares in North Africa and South Asia (Skovmand et al., 1997). This breadth of adaptation, achieved via testing of breeding materials in a very extensive sampling of the TPE during selection, resulted in increased tolerance to a range of naturally-occurring stresses. In the US Corn Belt, wide-area testing was shown to have increased tolerance to stresses such as drought, low fertility, and cold (Castleberry et al., 1984, Duvick, 1997, Tollenaar et al., 2000). Similarly, a wide-area testing network was important within CIMMYT's maize breeding program when selecting for tolerance to heat stress (Cairns et al., 2013b).

Multi-environment trials (METs) are expensive and complex to conduct on the scale needed to provide information useful in predicting cultivar adaptation to climate change; only the largest regional and international breeding programs have the capacity to implement them. Small breeding programs in developing countries often are unable to conduct METs properly sampling their own TPE, let alone related environments outside their TPE useful in predicting responses to a future climate. Better access to information on cultivar performance in broad-scale multi-location trials, regionally coordinated (usually by CGIAR centers) will help small national breeding programs accelerate adaptation to climate change.

METs have been complemented by other selection tools for tolerance to a broad range of stresses. Several programs have used alternating selection in contrasting seasons, either at the same location or at different sites, to expose materials to a broad range of potential production environments. One notable example is the shuttle breeding system used for half a century in the CIMMYT wheat breeding program, wherein nurseries are conducted at cool, high-elevation locations in the Mexico City area during the dry season and at the much hotter and drier Ciudad Obregon site in the wet season (Trethowan et al., 2007). Another is selection in both the wet and dry seasons at Los Banos in the Philippines in the IRRI irrigated rice breeding program (Wassmann et al., 2009). Screening under managed drought stress, wherein materials are evaluated under low-rainfall conditions, with irrigation withheld for critical crop stages, has been used extensively in maize and rice breeding and genetic analysis, contributing to the development of stress tolerant cultivars (Banziger et al., 2006) and identification of quantitative trait loci (QTL) affecting drought tolerance (Bernier et al., 2007).

### 2.4 The need for seed systems that rapidly replace varieties

Even if breeding programs obtain heat- and drought-tolerant parents, aggressively accelerate breeding cycles, and test materials under the full range of environments likely to occur in the future within their TPE, they will not contribute to climate change adaptation if varieties are not continuously replaced in farmers’ fields. Commercial breeding programs in temperate cropping systems, notably in the US Corn Belt, can deliver climate change adaptation through rapid varietal replacement. In the US, it takes about 6 years to develop and deploy a new maize hybrid, which then remains in use for an average of only three to four years before it is replaced (Brooks, 2009). As a result, farmers are always using varieties that have been bred in the present conditions. This rapid-replacement model arose out of intense competition among seed companies for market share. Similar trends have been observed in other temperate regions, including Europe and China. Rapid adaptation to climate change is an unintended benefit of this system. Commercial farmers in temperate regions make data-driven cultivar choices, and replace varieties even for a very small potential yield increase. They have a high degree of confidence in the data provided by seed companies and extension services. Companies thus have a strong incentive to maximize rates of genetic gain, and to disseminate a steady stream of improved products. Matching the effectiveness of such systems in delivering climate change adaptation is a critical challenge for the public sector breeding and seed systems that serve most farmers in the developing world.

Brennan and Byerlee (1991) proposed the *average area-weighted age of varieties in farmers’ fields* as a measure of the rate of varietal replacement on-farm. Reliable estimates of this important parameter are rare; a few are summarized in Table 2. In many regions, farmers continue to grow varieties that were developed early in the Green Revolution; this slow rate of varietal turnover is likely contributing to the yield stagnation reported in many parts of the world by Ray et al. (2012). To take just a few examples, over 25% of the South Asian wet-season rice area is still planted to the variety Swarna, which was released about 35 years ago (IRRI, unpublished data). In India, the average age of wheat varieties in farmers' fields increased from 9 years in 1997-8 to 13 in 2007-8 (Krishna et al., 2014). Because the time it takes to develop and release a wheat variety via pedigree breeding in India is 10–15 years, this means most Indian farmers are using varieties selected over 20 years ago. Smale et al. (2008) showed that slow varietal replacement was retarding wheat productivity growth in Punjab, India. The situation is similar throughout the developing world; hundreds of millions of farmers are growing landraces or older improved varieties that are not optimized for today's climate or production systems. By growing older varieties, farmers are also missing out on the benefits of many years of genetic gains from the breeding programs that serve them, but that are insufficiently linked to them as a result of dysfunctional varietal release and seed systems.

**Table 2:** Average age of varieties in farmers’ fields for several crops and countries.

**Crop**	**Country**	**Average age of variety (years)**	**Weighting basis**	**Year of estimate**	**Source**
Hybrid maize	US	3	Area	2016	Brooks (2009)
Hybrid maize	Kenya	17	Area	2010	Smale and Olawande (2014)
Rainfed rice	India	28	Area	2014	IRRI, unpublished data
Wheat	India	13	Seed production	2008	Krishna et al. (2014)

Slow varietal turnover in Sub-Saharan Africa and South Asia is likely to result in large part from lack of information about and access to new varieties on the part of smallholder farmers. It may also result from failure of new varieties to match old ones in key quality or other characteristics even when they exceed them in yield potential, disease resistance, or stress tolerance. However, there are many cases of rapid adoption of new varieties in smallholder agriculture when new varieties offer clear and significant advantages over currently-used varieties, and when farmers have access to seed.

In pure line crops where farmers can plant saved seed, the evolution of competitive seed markets driving rapid varietal replacement is slow in the absence of hybrid or transgenic products because of the lack of a business model that provides sufficient revenue to seed producers to support their own breeding programs. In most of these cropping systems, which cover much of the developing world, the breeding and dissemination of new varieties will therefore remain a public sector responsibility for many years to come. It is therefore critical that public systems recognize the need for faster varietal replacement. But even in systems where seed purchase rates are high, as in hybrid maize in Eastern and Southern Africa, varietal replacement can be extremely slow when there is little competition among seed companies. Examples include the Kenyan highlands, where hybrids developed in the 1970s and 1980s are still in use, and much of Southern Africa outside of South Africa, where 15-year-old hybrids like SC513 are still widely grown despite the existence of much more productive varieties. Experience in Africa shows that high rates of hybrid seed penetration are no guarantee of rapid hybrid turnover.

Old cultivars remain in use too long for a variety of reasons that need further study, but several problems seem obvious. A key factor in slow rates of varietal replacement is certainly the degree of commercialization of the cropping system (Spielman and Smale, 2016); farmers in cropping systems characterized by low market linkage and production mainly for subsistence purchase few inputs, and are unlikely to replace varieties rapidly unless a new pest or disease makes their preferred traditional varieties unusable. Continuous, rapid varietal turnover, the precondition for plant breeding to contribute effectively to climate change adaptation, is likely only sustainable in commercialized cropping systems where farmers frequently purchase seed. Although one-off public-sector dissemination campaigns have occasionally been successful in driving adoption of new varieties (e.g. the Green Revolution in wheat and rice in South Asia), they rarely lead to a culture of rapid varietal turnover. In public seed systems, government seed corporations may not be incentivized for rapid varietal turnover; it is simpler and less expensive to produce and distribute seed of a currently popular variety than to incur the costs of its replacement. Likewise, in a private sector system where there is not intense competition, companies have little incentive to substitute a currently popular variety with a newer and more productive one. Changing varieties entails heavy costs for a seed company. Especially in hybrid crops like maize, companies face a steep learning curve in bringing a new variety into production. Seed companies often claim that they market obsolete varieties because farmers ask for them, but in this chicken-and-egg scenario, farmers request old varieties because they do not know that better ones are available. New varieties must be heavily advertised and promoted to familiarize farmers with their advantages. Millers and processors must be convinced that the new variety will serve their needs. Seed companies will not incur promotional costs unless they are being “chased” by competitors. Seed producing organizations, whether public or private, must be incentivized or induced to withdraw an old variety from production when a superior new one becomes available, even if farmers continue to request the old one by name because of habit.

Ministries of agriculture, variety release systems, and government seed companies can speed up varietal turnover by:

1. Clearly identifying the new varieties they recommend, describing their advantages over the variety they are replacing, supported by reliable data;
2. Aggressively demonstrating and promoting these varieties
3. De-certification of obsolete varieties when they are superseded by better ones;
4. Withdrawal of seed subsidies for obsolete varieties;
5. Withdrawing funds from the production of breeder and foundation seed of obsolete varieties
6. Setting targets for the average varietal age in foundation seed production and in farmers’ fields
7. Simplifying and harmonizing variety release processes regionally to build private sector confidence and participation in the seed sector

A key element in generating a culture of rapid varietal replacement is convincing farmers that it is in their interest to change varieties as soon as a new one is endorsed and made available by the seed system. After the initial changeover from traditional to improved varieties, which tend to bring large increases in yield that are obvious to the naked eye, subsequent new varieties may not be sufficiently improved such that their advantages are detectable without data from replicated multi-environment trials (METs). A single cycle of varietal substitution may give a benefit of only 5–6% (the gains that can be expected from three or four years of highly effective breeding) over the previous variety, an advantage that cannot be visually discerned by farmers but can be detected through METs. However, over several breeding cycles, the benefit of aggressively adopting new varieties is large. Farmers need enough trust in both the products and the information provided about them to ensure that they demand improved varieties based on data and recommendations provided by reliable advisers, rather than just on visual demonstration. Dissemination models that rely on farmer-to-farmer spread of improved varieties based on farmer demand generated by visible superiority over the current dominant variety in demonstration plots simply do not work after the initial adoption of improved varieties, unless the new variety is tolerant to a serious disease or pest affecting the old one. In the high-functioning seed systems that offer farmers the best protection from climate change, new varieties are pushed into farmers’ fields, not pulled.

## 3 Conclusions

We have argued here that climate change adaptation in crop production can be delivered by rapid-cycle breeding programs that generate a steadily improving stream of varieties. In most breeding programs in the developing world, breeding cycles are at least twice as long as they should be, and could be significantly reduced with modest investment in rapid generation advance, irrigation, genomic prediction, and recycling of new parents on the basis of field testing protocols whose duration is optimized for breeding value rather than the release of finished varieties.

If such programs are to deliver rapid climate change adaptation, it is critical that they have access to elite germplasm from other regions already experiencing the “future climate”. Such access has become increasingly restricted, both due to the enforcement of strong forms of intellectual property protection on plant varieties in some jurisdictions and to restrictions imposed by some on the exchange or export of germplasm considered to be proprietary or part of a national genetic patrimony. Climate-adaptive breeding systems must also test potential new varieties in many locations, several seasons, and carefully-designed managed environments, to ensure that they are challenged by the range of environmental conditions they will encounter in farmers’ fields.

As critical as the breeding systems are seed systems that continuously replace varieties, ensuring that farmers are always using varieties selected in the current climate. The goal of national seed systems in the developing world should be to ensure that the average age of varieties in farmers’ fields is under 10 years, both to ensure that genetic gains are delivered steadily to farmers and to keep pace with the effects of climate change. Breeding organizations, regulatory bodies responsible for varietal release, national seed systems, and seed companies need to take responsibility for increasing the rate of varietal turnover in farmers’ fields. Rapid-cycling seed systems are already in place in commercial temperate cropping systems with highly competitive seed markets, but the situation in the developing world is starkly different, with most farmers still using either landraces or varieties that were released over 20 years ago.

These key components of climate-adaptive breeding systems need to be strengthened and modernized in crop improvement organizations serving farmers in the developing world. A change in the mindset and organization of seed systems to emphasize rapid and continual varietal replacement is needed. Finally, the effectiveness of platforms for international germplasm exchange and regional testing, coordinated in large part by the CGIAR, must be enhanced, with the support of national governments, charitable foundations, and the private sector.
